# Designing New Chimeric Proline-Rich Antimicrobial Peptides to Enhance Efficacy Toward the ESKAPE+E: Beyond Sequence Extension

**DOI:** 10.3390/biom15060776

**Published:** 2025-05-27

**Authors:** Adriana Di Stasi, Luigi de Pascale, Martino Morici, Daniel N. Wilson, Marco Scocchi, Mario Mardirossian

**Affiliations:** 1 Department of Life Sciences, University of Trieste, 34127 Trieste, Italy; adriana.distasi@units.it (A.D.S.); luigi.depascale@phd.units.it (L.d.P.); 2 Institute for Biochemistry and Molecular Biology, University of Hamburg, 20146 Hamburg, Germany; martino.morici@uni-hamburg.de (M.M.); daniel.wilson@uni-hamburg.de (D.N.W.)

**Keywords:** drug design, antimicrobial peptide, proline-rich peptide, ESKAPE+E, protein synthesis, antibiotic resistance

## Abstract

Proline-rich antimicrobial peptides (PrAMPs) primarily exert their antimicrobial effects intracellularly, inhibiting protein synthesis. B7-005, a synthetic 16-amino acid PrAMP, has a broader antimicrobial spectrum compared to native counterparts, despite shorter PrAMPs typically exhibiting reduced activity. This study aimed to enhance B7-005’s potency by extending it with 6 or 11 amino acids derived from the C-terminal sequences of cetacean Tur1A and Lip1 PrAMPs, as well as bovine Bac7(1-35). Six chimeric derivatives were evaluated for antimicrobial and bactericidal potency, cytotoxicity, bacterial membrane permeabilization, and in vitro inhibition of protein synthesis. Extending B7-005 with sequences from other PrAMPs increased its activity against most ESKAPE+E pathogens, reducing minimum inhibitory concentration (MIC) values by 2- to 8-fold, with notable differences among bacterial species, without increasing cytotoxicity toward the A549 cell line. All chimeras retained the ability to inhibit protein synthesis in *Escherichia coli* and to modestly perturb the *E. coli* membranes like B7-005. These novel chimeric PrAMPs, particularly the 22-mer derivatives, hold promise for developing new antimicrobial agents. The study also highlights variability in bacterial responses to PrAMPs and underscores how minor sequence differences can significantly impact efficacy against specific microorganisms. PrAMPs thus represent a valuable scaffold to rationally design derivatives targeting high-priority pathogens.

## 1. Introduction

Antimicrobial peptides (AMPs) are small, bioactive proteins naturally produced by organisms across all three domains of life [1]. In prokaryotes, AMPs function as competitive inhibitors against other microorganisms [2], while in eukaryotes, they are essential components of the innate immune system, directly killing pathogens or modulating immune responses [3]. The rising interest in AMPs as potential therapeutic agents [4] is driven by the global challenges of antibiotic resistance and re-emerging infectious diseases [5,6]. Developing alternatives to traditional antibiotics could significantly enhance public health, improve healthcare systems, and yield economic benefits.

Most AMPs are short, positively charged peptides composed of 10–50 amino acids, with a net positive charge ranging from +2 to +11 [3]. They are characterized by approximately 50% hydrophobic residues, essential for their interaction with bacterial membranes, their primary target [7].

Proline-rich antimicrobial peptides (PrAMPs) are a distinct subclass of AMPs, found in arthropods and mammals, with unique features. These peptides are less hydrophobic than typical AMPs, rich in proline and arginine residues and primarily act intracellularly as protein synthesis inhibitors [8]. At low active concentrations, PrAMPs are internalized into susceptible Gram-negative bacteria via the inner-membrane transport protein SbmA/BacA and secondarily Yjil/MdtM [9]. Once in the cytosol, PrAMP target the bacterial translational machinery by binding the ribosomes at level of exit tunnel and blocking the elongation (e.g., Onc112 and Bac7 fragments) [9] or the termination (e.g., Api137 [10] and Drosocin [11]) step of the protein synthesis. This specific uptake mechanism contributes to their narrower spectrum of activity compared to membrane-targeting AMPs, as they rely on these transporters, which are unevenly distributed among bacterial species [9].

Bac7 is a natural PrAMP isolated from the large granules of bovine neutrophiles [12]. Its fragment encompassing the first 35 N-terminal residues of its sequence, Bac7(1-35), was the first mammalian PrAMP demonstrated to inhibit protein synthesis [13] Efforts to optimize Bac7(1-35) have included further shortening of the sequence that identified Bac7(1-16) as its shortest active fragment significant antimicrobial activity [14]. This short fragment retained also the ability of the longer Bac7(1-35) to inhibit protein synthesis [15], although its spectrum was limited to SbmA/BacA-producing species. With the aim to potentiate the antimicrobial activity of Bac7(1-16), this peptide underwent further optimization processes like systematic sequence modification [16]. This latter approach led to the development of B7-005. This 16-amino acid artificial derivative of Bac7(1-16) has a broadened spectrum of activity encompassing also Gram-positive bacteria and most ESKAPE pathogens [16] while still remaining not cytotoxic [17]. ESKAPE is the collective definition for a group of microorganism of major concern according to the WHO due to antibiotic resistance phenomena (*Enterococcus faecium*, *Staphylococcus aureus*, *Klebsiella pneumoniae*, *Acinetobacter baumannii*, *Pseudomonas aeruginosa*, and *Enterobacter species*) [18,19]. In the light of this situation, the broader antimicrobial spectrum of B7-005 with respect to Bac7(1-16) is therefore desirable and it is attributed to its higher hydrophobicity, which improves its affinity for the bacterial membranes. This feature provides B7-005 with a mechanism for internalization in bacterial cells largely independent of SbmA/BacA [16]. The minor dependency of B7-005 on active transport systems limits the development of resistance in *E. coli* [17] since the mutation or loss of the transport protein is insufficient to strongly decrease the bacterial sensitivity to this peptide [16]. However, the mode of action of B7-005 is complex and flexible. The peptide targets all the ESKAPE pathogens, although with diverse antimicrobial efficacy. Similarly, the efficacy of B7-005 in inhibiting the ribosomal activity in vitro also varied among ESKAPE microorganisms. For most of the ESKAPE the inhibition of the protein synthesis, in fact, probably concurs to various extents to the antimicrobial mechanism of B7-005, which instead relies also, and predominantly, on the destabilization of the bacterial membrane [20]. Anyhow, B7-005 probably suffers from the limitations of shortened PrAMPs that often exhibit valuable antimicrobial potency but at lower levels than that of their longer native counterparts. This, nevertheless, suggests potential for further optimization by re-elongating the already optimized shorter PrAMPs. The cetacean PrAMPs Tur1A, Bal1, and Lip1, are homologous to Bac7(1-35) and, similarly to their bovine orthologue, they are inhibitors of the protein synthesis [21]. While they share high sequence similarity in the N-terminal 18 amino acids, their C-terminal regions diverge significantly from that of Bac7(1-35) [21]. Since cetacean PrAMPs exhibit a broader spectrum of activity compared to Bac7(1-35), this property is likely attributable to their divergent C-terminal sequences. Consequently, these C-terminal sequences could serve as valuable tools to enhance the functionality of existing PrAMPs.

This study aimed to enhance the potency and range of efficacy of the 16-amino acid B7-005 peptide by extending it with 6 or 11 amino acids from the C-terminal sequences of the cetacean Tur1A and Lip1, as well as from the bovine Bac7(1-35) PrAMPs. Six hybrid derivatives were generated and evaluated for their antimicrobial potency, killing efficiency, and cytotoxicity. Their mechanism of action was also investigated, focusing on bacterial membrane permeabilization and in vitro inhibition of protein synthesis. The extension of B7-005 with sequences from other PrAMPs led to a differential and species-specific improvement of their antimicrobial properties against both Gram-positive and Gram-negative bacteria. These results offer valuable insights for designing more effective antimicrobial agents.

## 2. Materials and Methods

### 2.1. Peptides

All peptides were synthesized by NovoPro Bioscience (Shanghai, China) using solid-phase F-moc chemistry, purified by RP-HPLC to >95% purity, and their molecular weight was confirmed via mass spectrometry. The peptides were shipped lyophilized. Upon arrival, peptides were resuspended in 500 μL of 10 mM HCl and lyophilized three times to replace TFA with a less toxic chloride counterion. The peptides were then dissolved in sterile milli-Q water and quantified using absorbance at 214 nm and 280 nm based on Lambert–Beer’s law. The molar extinction coefficient of peptides at 214 nm was calculated according to Kuipers and Gruppen [22] with small modifications. The peptides were stored at −20 °C.

### 2.2. Solubility Test

The solubility of the peptides in Müller–Hinton broth (MHB) (Difco Inc., Becton Dickinson, Sparks, MD, USA) was assessed by measuring any increase in the optical density (OD) over time at 600 nm, using the Nanoquant Infinite-M200Pro plate reader (Tecan Trading AG, Switzerland). Peptides diluted to several concentrations in MHB were incubated in a 96-well plate (Sarstedt, Milan, Italy) at 37 °C, and OD was measured at 0, 4, and 24 h. Results are presented as the mean ± standard deviation (SD) of three independent experiments (*n* = 3).

### 2.3. Bacterial Cultures

Reference bacterial strains, including *Escherichia coli* BW25113, *Escherichia coli* ATCC 25922, *Klebsiella pneumoniae* ATCC 700603, *Acinetobacter baumannii* ATCC 19606, *Enterobacter cloacae* ATCC 13047, *Pseudomonas aeruginosa* ATCC 27853, *Staphylococcus aureus* ATCC 25923, and *Enterococcus faecium* ATCC 19434, were obtained from the American Type Culture Collection (ATCC) in Manassas, VA, USA. Strains were stored at −80 °C in glycerol stocks until use. For all experiments, cultures were grown in sterile MHB (Difco Inc., Becton Dickinson, Sparks, MD, USA). Overnight cultures were incubated at 37 °C for approximately 18 h under shacking (140 rpm, then diluted 1:30 in fresh MHB and again shaken at 140 rpm for ~2 h to reach the mid-log phase (OD ~0.3 at 600 nm). The bacterial cultures were then diluted to the desired concentration for experiments. *E. coli* BW25113 Δ*sbmA* (Keio collection [23]) was grown in MHB with the addition of 50 μg/mL kanamycin (Sigma, Milan, Italy).

### 2.4. Antimicrobial Activity

Antimicrobial activity was assessed using the Minimum Inhibitory Concentration (MIC) assay, following CLSI guidelines. Briefly, peptides were serially diluted in MHB across a 96-well round-bottom plate (Sarstedt, Milan, Italy), starting with 50 μL in the first well. A mid-log bacterial culture was diluted to 5 × 10^5^ CFU/mL, and 50 μL of this suspension was added to each well (except the MHB sterility control wells). Plates were incubated at 37 °C for 18 h, and the MIC was defined as the lowest peptide concentration that completely inhibited visible bacterial growth. MIC values were confirmed by at least three independent experiments (*n* ≥ 3).

The Minimal Bactericidal Concentration (MBC) was determined by plating 25 μL from clear MIC wells onto MH-agar Petri dishes (Difco Inc., Becton Dickinson, Sparks, MD, USA), followed by overnight incubation at 37 °C. The MBC was the lowest compound concentration, reducing bacterial viability by ≥99.9%. Results were based on at least three independent experiments (*n* ≥ 3).

### 2.5. Analysis of Bacterial Membrane Integrity

Bacterial membrane integrity was analyzed using flow cytometry (Attune NxT^®^, ThermoFisher, Waltham, MA, USA) by measuring propidium iodide (PI) uptake, with permeabilization expressed as the percentage of PI-positive cells. Bacterial cultures of *E. coli* ATCC 25922 (2.5 × 10^5^ CFU/mL) were incubated at 37 °C for 30 min with peptides at ½ MIC, MIC, and 2 × MIC concentrations. PI (10 μg/mL) was added 5 min before analysis. Colistin at ½ MIC, MIC, and 2 × MIC served as a positive control, while water replaced the peptide in the negative controls. Data were collected using Attune NxT software and presented as means ± SD from three independent experiments (*n* = 3).

### 2.6. In Vitro Translation Assays

To assess translation inhibition by the compounds in this study, an *E. coli* cell-free lysate-based translation system (RTS100 *E. coli* HY; biotechrabbit) was employed for firefly luciferase (fluc) expression, as previously described for other translation-inhibiting antibiotics [24]. Briefly, 5 μL reactions were prepared according to manufacturer protocol, mixed with 1 μL of the tested compound at the indicated final concentrations. Each reaction mix also contained purified fluc mRNA (16 ng/μL), whose translation product was exploited as a reporter. The reactions were incubated for 30 min at 32 °C at 600 rpm and were stopped by adding 3 μL of kanamycin (50 μg/μL) to each reaction tube. The stopped reactions were transferred to a black 96-well chimney flat-bottom microtiter plate and mixed with 40 μL of fluc substrate (Promega, Madison, WI, USA). Luminescence was measured using a TECAN infinite 200Pro plate reader (Tecan Trading AG, Switzerland). Samples were normalized relative to reactions without antibiotics.

### 2.7. In Vitro Cytotoxicity Assay

The human lung carcinoma epithelial cell line (A549) was obtained from the American Type Culture Collection (ATCC) (Manassas, VA, USA). A549 cells were cultured in adhesion flasks (EuroClone, Milan, Italy) with complete Dulbecco’s MEM (DMEM, EuroClone, Milan, Italy) containing high glucose, supplemented with 100 U/mL penicillin, 100 mg/mL streptomycin (Sigma, Milan, Italy), 2 mM L-glutamine, and 10% FBS (Life Technologies, Carlsbad, CA, USA). Cells were incubated at 37 °C with 5% CO_2_.

Cytotoxicity in A549 cells was assessed using the MTT assay. After reaching confluence, cells were counted using a Bürker–Türk chamber and diluted to 2 × 10^5^ cells/mL in DMEM. A total of 100 μL of this suspension was seeded into 96-well plates (Sarstedt, Milan, Italy) and incubated overnight at 37 °C with 5% CO_2_. The next day, the spent medium was replaced with 100 μL of fresh DMEM medium containing peptide or water at the desired concentration. After 20 h of incubation, 25 μL of 1 mg/mL MTT (Sigma, Milan, Italy) in PBS was added, and the plates were incubated for 4 h in the dark. The solution was then removed, wells washed with PBS, and 100 μL of IGEPAL (10% *w*/*v* in 10 mM HCl) (Sigma, Milan, Italy) was added to dissolve the MTT crystals. After overnight incubation, absorbance was measured at 570 nm using a Nanoquant Infinite-M200Pro plate reader (Tecan Trading AG, Switzerland). The cytotoxicity was calculated by comparing the OD of treated samples with that of untreated controls. The results are the average ± SD of at least three independent experiments in internal triplicate (*n* = 9).

### 2.8. Statistical Analysis

To assess significant differences between the experimental groups, a one-way ANOVA followed by Dunnett’s multiple comparisons test or a two-way ANOVA followed by Tukey’s multiple comparisons test was performed. A *p*-value of ≤0.05 was considered statistically significant. The software GraphPad 10 was used for statistical tests.

## 3. Results

### 3.1. Design and Characteristics of Chimeric Peptides

To design peptides potentially endowed with stronger antimicrobial activity than B7-005 but, on the other hand, characterized by short sequences (simplifying synthesis procedures), 6- and 11-amino acid residue extensions were appended to the already rationally modified PrAMP B7-005. The 16-amino acid peptide was extended by adding C-terminal fragments derived from the internal sequences of three natural mammalian PrAMPs, Bac7(1-35), Tur1A and Lip1, selected for their antimicrobial potency and activity spectrum. Specifically, we chose 6- and 11-amino acid fragments from the 17th position onward (i.e., residues 17–22 and 17–27) of Bac7(1–35) to design B7-B6 and B7-B11, respectively. Similarly, we selected the residues 17–22 and 17–27 of the Tur1A to obtain B7-T6 and B7-T11 and the corresponding fragments from Lip1 to create B7-L6 and B7-L11 (Table 1).

The extensions were decided to maintain the overall chemico-physical properties conferred by the residues but modify their position. Moreover, when possible, a C-terminal Arg residue was granted to help solubility and discourage aggregation among lipophilic moieties. In fact, the overall proline content and the net positive charge were maintained consistently among the six chimeras (Table 1). All six chimeras exhibited a lower percentage of positively charged arginines (37–40 versus 50% in B7-005) and a higher percentage of proline residues (27–33% versus 18.8% in B7-005). Also, all chimeras were slightly more hydrophobic than B7-005, with the 11-amino acid-extended peptides B7-T11 and B7-L11 being the more hydrophobic derivatives (Table 1).

Some derivatives had solubility issues when dissolved in Mueller–Hinton broth (MH) at high concentrations. To assess this, we evaluated the tendency of each peptide to precipitate in MH by scattering measurements. We observed that, in a concentration-dependent manner, some peptides tended to aggregate at concentrations between 32 and 64 µM, particularly Lip-derived B7-L6 and B7-L11. However, the phenomenon diminished over time (within 24 h; Figure S1) and did not occur in PBS and in the cell culture medium DMEM (data were negative, not shown here). Consequently, the concentrations of B7-L6 and B7-L11 used in the MIC assays were prudently kept below 64 µM.

### 3.2. Antimicrobial Activity Against ESKAPE+E Pathogens

The antimicrobial activity of the chimeric peptides was tested toward representative ATCC reference strains of each of the ESKAPE pathogens, as well as of *Escherichia coli*, in full MH broth. Moreover, to get hints on the mode of action of the new peptides, their activity was also evaluated against an *E. coli* strain lacking the membrane transporter SbmA. Minimum inhibitory and bactericidal concentrations (MIC&MBC) were determined (Table 2, compound names are reported without the B7- suffix). In most cases, the chimeric peptides exhibited increased antimicrobial activity compared to the native B7-005. The improvement of antibacterial potency varied according to both the sequence composition and the length of the extension added to B7-005. At least one new derivative displayed a four-fold increase in antimicrobial activity compared with the native B7-005 toward every tested pathogen (Table 2). The only exceptions were the two strains of *E. coli* that were affected similarly by the chimeras and *K. pneumoniae* that displayed the same sensitivity toward all the tested compounds. Specifically, the chimeras B7-B6 and B7-B11 demonstrated a four-fold increase in activity compared to B7-005 against *A. baumannii* and *E. cloacae* and an enhanced activity also against Gram-positive *E. faecium* and *S. aureus*. Similarly, B7-T6 and B7-T11 exhibited a four-fold improvement in efficiency against *E. cloacae* and *A. baumannii.* In addition, B7-T11 showed an eight-fold increase in activity against *E. faecium*.

Lastly, the derivatives B7-L6 and B7-L11 displayed the greatest improvements, with an eight-fold increase against *E. faecium* and a four- to eight-fold increase against *E. cloacae*.

In contrast to the -B and -T derivatives, these compounds also demonstrated enhanced activity (four-fold) against *P. aeruginosa*. Notably, B7-L11 exhibited very high activity also against *S. aureus* (MIC = 4 µM). Interestingly, in all cases, the MBC values were equal to or only two-fold higher than the MIC values, suggesting that all peptides exert a bactericidal effect. The only exception was observed with *E. faecium*, which exhibited MBC values four- to eight-fold higher than the MIC (Table 2).

All the chimeras, as previously observed in B7-005 [16], displayed little or no dependence on the SbmA transporter (Table 2), suggesting that they, like B7-005, may share a similar or identical mode of internalization into the cells, at least in *E. coli* cells.

### 3.3. Cytotoxicity Against Human Cell Line A549

**Figure 1:**
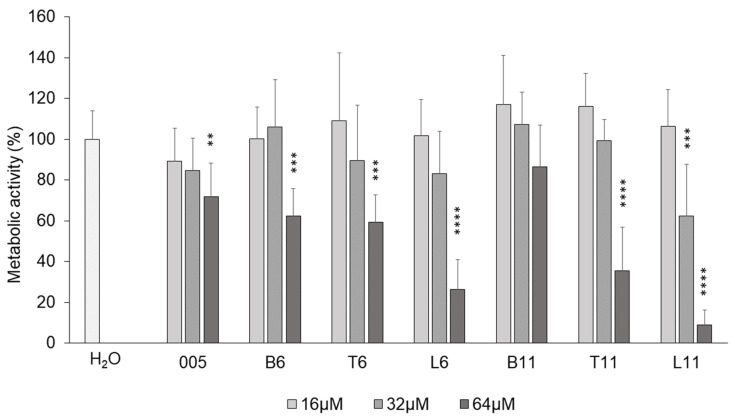
Viability of A549 cells measured by MTT assay in the presence of the peptides at different concentrations. MTT assay on A549 cells after treatment with different concentrations of peptides. The cell viability was measured (as absorbance at 570 nm) after 24 h from the exposure to the peptides. Samples treated with water instead of peptides were set as 100% of viability (H_2_O). Error bars are the standard deviations calculated on the average of at least three independent experiments performed as internal triplicates (*n* ≥ 9). ** *p* ≤ 0.005 vs. H_2_O; *** *p* ≤ 0.0005 vs. H_2_O; **** *p* < 0.0001 vs. H_2_O (one-way ANOVA followed by Dunnett’s multiple comparisons test).

To assess whether the different extensions of B7-005 affected the biocompatibility of the new peptides with eukaryotic cells, the human pulmonary cell line A549 was selected as a model to compare the effect of the extension among the new peptides. A549 cells were therefore exposed to an increasing concentration of each peptide for 24 h, and their viability was then evaluated using the MTT assay. None of the peptides exhibited cytotoxic effects up to 32 µM, except for B7-L11, which also showed toxicity at this concentration. In contrast, B7-B11 did not show any toxicity, even at 64 µM (Figure 1). Overall, increasing the peptide length *per se* did not markedly decrease the biocompatibility of the peptides. Instead, cell viability was affected by the composition of the extensions.

### 3.4. Interaction with the Bacterial Membrane

**Figure 2:**
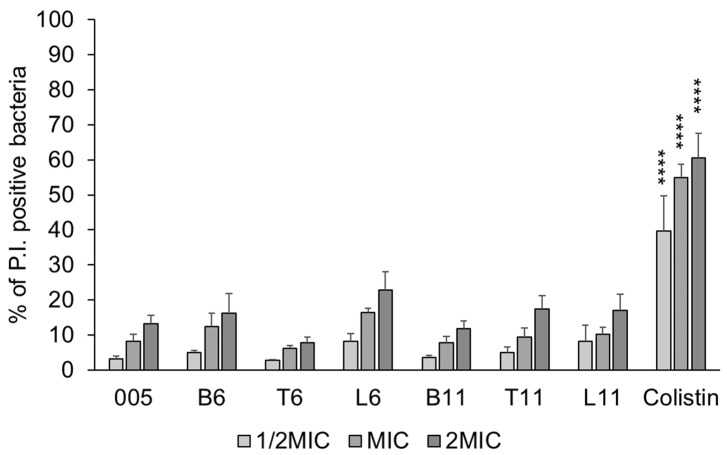
Membrane-damaging activity of the chimeric peptides on *E. coli* ATCC 25922. The original B7-005 and the lytic antibiotic colistin were used as comparisons. The permeabilization assay was performed in MHB, treating for 30 min 2.5 × 10^5^ CFU/mL bacteria with the compounds at ½ MIC, MIC, and 2 × MIC. The % of PI-fluorescent bacterial cells over the whole population was measured. Data are the average ± SD of three independent experiments (*n* = 3). **** = *p* < 0.0001 vs. B7-005 (two-way ANOVA followed by Tukey’s multiple comparisons test).

Given the higher hydrophobicity of the chimeric peptides with respect to the B7-005, we investigated whether the new compounds exhibited enhanced membrane destabilization activity. To this aim, membrane permeabilization was evaluated by assessing the propidium iodide (PI) uptake in *E. coli* cells, chosen as a bacterial model. Overall, at both MIC and 2 × MIC, all peptides maintained a low membrane-destabilizing level (PI+ cells < 10–15%), not significantly different from B7-005. Notably, all the chimeric peptides displayed a permeabilization profile markedly different from that of the membranolytic antibiotic colistin, which was used as a reference (Figure 2), suggesting moderate destabilization of the *E. coli* membrane by these compounds.

### 3.5. Inhibition of Protein Synthesis

**Figure 3:**
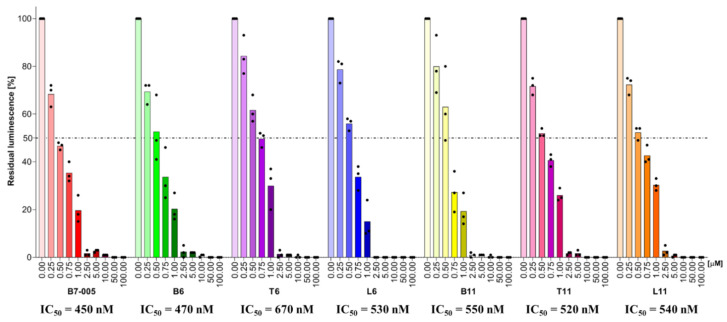
Inhibition of in vitro protein synthesis by B7-005 and chimeric peptides on commercial *E. coli* lysates. The inhibitory effect was evaluated as the residual luminescence signal (%) as a result of the translation of the reporter firefly luciferase. Controls received water instead of peptide and were set as 100% luciferase production. Histograms represent the mean of independent triplicates (*n* ≥ 3), whose single results are reported as black dots. The dashed line indicates 50% residual luminescence.

B7-005 is an efficient inhibitor of protein synthesis. To ensure that the sequence extensions did not compromise this important feature, the capability of the chimeric peptides to inhibit bacterial synthesis was checked using an in vitro translation assay with *E. coli* lysates. In vitro protein synthesis reactions were performed in the presence of the new peptides and of B7-005 as well to assess their capability to prevent the translation of a synthetic mRNA encoding firefly luciferase. Despite differences in length and composition of the C-terminal extensions appended to B7-005, all chimeric peptides showed very similar IC_50_, indicating that they maintained the same concentration-dependent inhibitory effect of the original B7-005 toward the *E. coli* translational machinery (Figure 3).

## 4. Discussion

Previous studies have identified B7-005 as the best derivative among hundreds of 16-amino acid PrAMP analogues due to its broad-spectrum activity and high biocompatibility [16], despite being one of the shortest, although designed, PrAMPs [8]. To evaluate whether prolonging B7-005 could enhance its antimicrobial efficacy while preserving its favorable properties regarding the mode of action and safety, we assessed the antimicrobial activity, biocompatibility, and mode of action of chimeric molecules generated by extending PrAMP B7-005 using sequence stretches from other native mammalian PrAMPs already characterized.

Results of this study provided evidence that the chimeric molecules increase the activity with respect to B7-005 against most ESKAPE pathogens, lowering the MIC values by two- to eight-fold, though with notable differences among the different bacterial species. However, at least one chimera proved to have a greater antimicrobial activity than B7-005 toward at least each of the eight strains tested, with the only exceptions being *E. coli* and *K. pneumoniae,* which remained invariably affected. In this sense, the extension of B7-005 can be considered successful. This result aligns with the fact that most natural PrAMPs are relatively long, with sequences rarely shorter than 19–20 residues and often exceeding 30 residues [8]. One may argue that, for RW-peptides, much shorter sequence length it is sufficient to get the maximum antimicrobial activity [8]. However, on the other hand, we think that the presence of WR-residues in these peptide is not sufficient to determine their mode of action as WR-peptides, but it may just influence or slightly modify their antimicrobial mechanism as PrAMPs. Extending B7-005 to 22 amino acidic residues re-establishes a length comparable to that of several natural PrAMPs. This sequence length could be important to guarantee the efficient inhibition of protein synthesis, as reported for the fragments of the PrAMP Bac5(1-25) [25] and/or to cross efficiently the membrane barrier [8]. On the other hand, quite surprisingly, the mere increase in length from 6 to 11 residues in most cases did not result in a further predictable enhancement of antimicrobial activity.

Analyzing the activity of the single chimeric compounds, no unique trend emerged as a definitive strategy for enhancing PrAMPs potency. Among the tested compounds, we noticed the chimeras B7-B6 and B7-B11, as well as B7-T6 and B7-T11, displayed increased activity compared to B7-005 against the Gram-negative *A. baumannii* and *E. cloacae,* as well as against the Gram-positive *E. faecium* and *S. aureus*. Notably, all the chimeric peptides exhibited no cytotoxicity at concentrations up to 32 µM, while B7-B11 remained non-cytotoxic even at 64 µM. Differently, B7-T11, which showed significantly enhanced activity against *E. faecium,* appeared to be cytotoxic at 64 µM. Since *K. pneumoniae* and *E. coli* are no more susceptible to these four derivatives than to the shorter B7-005, this suggests that the six-residue extension is specifically important for affecting *A. baumannii, E. cloacae*, *E. faecium,* and *S. aureus*. However, we cannot exclude that factors other than sequence length contribute to the increased activity of the B7-BX and B7-TX peptides against these four strains. In fact, these derivatives exhibit a higher percentage of proline residues (27–33%) compared to B7-005 (18.8%) and display greater hydrophobicity, as indicated by the GRAVY index values (−1.59 to −1.97 vs. −2.20).

The derivatives B7-L6 and B7-L11, in addition to demonstrating improved activity against *A. baumannii, E. cloacae*, *E. faecium*, and *S. aureus,* also exhibited enhanced potency against *P. aeruginosa*. The only peculiar feature of the B7-L series is the presence of a tryptophan (Trp) residue in position 18. The hypothesis that Trp may be critical for specifically targeting these species is supported by previous findings. The simple substitution of threonine (Thr) with Trp in the middle portion of an AMP significantly enhances it activity against *P. aeruginosa*, and this enhancement of activity was species-specific [26]. In another study, Trp end-tagged AMPs exhibited increased potency against *P. aeruginosa* [27]. Trp residues are found in high proportions in many AMPs and are consistently linked to essential functional roles in antimicrobial activity [28]. Although no clear mechanistic explanations exist for the species-specific activity of Trp enriched peptides against *P. aeruginosa* [26], our findings add valuable insights for AMP optimization and for the rational tailoring of antimicrobial agents targeting specific pathogens.

The 27-mer B7-T11 and B7-L11 exhibit the highest antimicrobial activity against *E. faecium.* These two derivatives are the most hydrophobic peptides according to the GRAVY index; therefore, it is plausible that hydrophobicity plays a role in enhancing their antimicrobial activity against *E. faecium*. This observation is in agreement with previous studies. Among some analogues of temporin-SHa with the same number of cationic residues, those displaying the highest activity against *E. faecium* were also the more hydrophobic [29]. Similarly, narrow-spectrum, highly potent, lactoferrin-derived peptides displayed selective activity against *E. faecium* [30]. These peptides are characterized by high hydrophobicity (58% of residues) and strong cationic properties (net charge +5). Since B7-005 is ineffective against *E. faecium* (Table 2 and previous publication [20]), the potent antimicrobial effect of B7-T11 and B7-L11 represents a significant achievement. Unfortunately, B7-L11 is the only peptide showing notable toxicity against the human A549 cell line at 32 µM, making it less selective than B7-T11. This result is not surprising, as several studies confirm that highly hydrophobic antimicrobial peptides lose selectivity for bacterial cells and become more toxic toward eukaryotic membranes [31,32].

To explain the differences in antimicrobial activity and cytotoxicity of the chimeric PrAMPs against various ESKAPE pathogens, we investigated their mechanism of action in *E. coli* cells. At least toward this microorganism, all the derivatives maintained a similar ability to inhibit protein synthesis in *E. coli*, comparable to that of B7-005. Furthermore, membrane permeabilization data confirmed that all chimeras had only a modest ability to interact with or perturb *E. coli* membranes, with the extent of membrane permeabilization being similar to that of B7-005. These findings suggest a low membrane-permeabilizing ability of the chimeras within short incubations, especially when compared with the well-known membranolytic peptide colistin. It is worth noting that the current data collected on *E. coli* cannot exclude a major (if not prominent) role of membranolysis in the mode of action of the chimeric peptides toward microorganisms different from *E. coli*, as has been previously observed for the unmodified B7-005 [20]. However, the combined data of protein synthesis inhibition and membrane permeabilization obtained in *E. coli* suggest that, regardless of the mode of action of the peptides against the specific pathogens, the extensions we have added to B7-005 are unlikely to drastically alter the molecular mechanism of the peptide, whatever it may be.

The various chimeras have antimicrobial activity against *E. coli* ATCC 25922 and *E. coli* BW 25113, which is no more than two-fold higher or lower than that of B7-005 (except for B7-L6 against *E. coli* ATCC 25922). Therefore, we conclude that the addition of C-terminal residues does not enhance the activity nor significantly alter the mechanism of action of the peptides toward *E. coli* and likely also *K. pneumoniae*. A peptide length of 16 residues appears sufficient to effectively kill these bacteria. On the other hand, the significant improvement of all the chimeras against *A. baumannii, E. cloacae*, *E. faecium,* and *S. aureus,* as well as for the B7-L series against *P. aeruginosa*, might be due to differences in their mechanisms of action. This hypothesis is in agreement with recent findings highlighting that B7-005 exhibits marked differences in its killing mechanisms across different ESKAPE species. Unlike in *E. coli* and *K. pneumoniae*, where B7-005 is internalized at its MIC (1–2 µM) without relevant membrane permeabilization, the targeting of the bacterial envelope appears to play a prominent role in *S. aureus*, *P. aeruginosa*, *E. cloacae*, *A. baumannii*, and possibly *E. faecium* [20]. This suggests that the chimeras may kill these ESKAPE pathogens through membrane interactions, as often reported also for non-proline rich AMPs [33], requiring them to be longer and/or more hydrophobic to achieve greater activity than B7-005.

We do not yet know from structures how these PrAMPs interact with ribosomes from different ESKAPE organisms, thus impairing proper structure–activity relationship considerations. Circular dichroism analysis of B7-005 suggested an uncommon scenario, possibly because of Pro, Trp, and Arg in pushing the structure toward a polyproline II helix or toward turns, depending on the environment [20]. This is also reminiscent of some aspects previously observed for Indolicidin [34]. The structural scenario for the chimeric peptides of the current study may become even more complex due to the extension comprising repeats of Pro, Arg, and also aromatic residues. Further focused studies may elucidate these aspects.

However, structural considerations apart, it is evident that most chimeras, especially the 22-mer derivatives, represent an improvement in antimicrobial activity over B7-005. Notably, B7-005 already exhibits a broader activity spectrum than the natural peptides Bac7(1-35) and Tur1A, which are completely ineffective against both *E. faecium* (MIC = 64 µM) and *S. aureus* (MIC > 64 µM) [21]. Therefore, chimeras such as B7-B6 and B7-B11, composed of B7-005 extended with a segment of Bac7(1-35), represent an improvement over both the native peptides, maintaining biocompatibility and SbmA independence. Similarly, B7-T6 and B7-T11, which extend B7-005 with a segment of Tur1A, demonstrated superior properties compared to natural Tur1A, despite the latter being longer than both B7-T6 and B7-T11. For B7-L6 and -L11, the benefits compared to Lip1, which is the donor of the sequence used to extend B7-005, are more limited [21]. This chimeric peptide exhibits improved antimicrobial activity only against *S. aureus*. However, it is worth noting that these chimeric peptides are shorter than Lip1 (22 and 27 residues vs. 32) and exhibit decreased cytotoxicity [35].

## 5. Conclusions

In conclusion, the design of these novel chimeric PrAMPs has resulted in promising lead compounds for the development of new antimicrobial drugs. This study also highlights the variability in bacterial species’ responses to the same PrAMPs and emphasizes how minor structural differences can significantly impact efficacy against the same microorganism. Further and systematic structure–activity relationship studies will be necessary on these (and also other) PrAMPs to clearly identify some aspects of their mode of action that may give valuable hints to the design of antimicrobial drugs. In fact, PrAMPs hold great potential for the rational design of derivatives that selectively target high-priority pathogens, which may contribute to the ongoing efforts to combat the antibiotic resistance crisis.

## Figures and Tables

**Table 1:** Sequences of the chimeric peptides designed for this study. The residues added to the B7-005 sequence are in bold.

Peptide	Sequence	Residues	% of Positive Charges	% Proline	GRAVY Index
B7-005	WRIRRRWPRLPRPRWR	16	50	18.8	−2.20
B7-B6	WRIRRRWPRLPRPRWR**PLPFPR**	22	41	27.3	−1.72
B7-T6	WRIRRRWPRLPRPRWR**PRFPPP**	22	41	31.8	−1.97
B7-L6	WRIRRRWPRLPRPRWR**PWFPPR**	22	41	27.3	−1.94
B7-B11	WRIRRRWPRLPRPRWR**PLPFPRPGPRP**	27	37	33.3	−1.76
B7-T11	WRIRRRWPRLPRPRWR**PRFPPPFPIPR**	27	37	33.3	−1.62
B7-L11	WRIRRRWPRLPRPRWR**PWFPPRFPIPR**	27	37	29.6	−1.59

**Table 2:** Antimicrobial activity of B7-005 and its derivatives. Representative strains of the ESKAPE+E were used, as well as *E. coli* BW25113 and *E. coli* BW25113Δ*sbmA*.

**Bacterial Strain**		**MIC (μM) ^§^**						
		**005 ^#^**	**B6**	**T6**	**L6**	**B11**	**T11**	**L11**
*E. coli*	ATCC 25922	1	2	0.5	4	1	2	2
*E. coli*	BW 25113	1	1	1	1	0.5	1	1
*E. coli*	BW 25113 Δ*sbmA*	2	2	1	2	1	1	2
*E. faecium*	ATCC 19434	32	8	16	4	16	4	4
*S. aureus*	ATCC 25923	16	8	16	8	16	16	4
*K. pneumoniae*	ATCC 700603	2	2	2	2	2	2	2
*A. baumannii*	ATCC 19606	4	1	1	2	1	1	2
*P. aeruginosa*	ATCC 27853	16	8	8	4	16	16	8
*E. cloacae*	ATCC 13047	8	2	2	2	2	2	2
**Bacterial Strain**		**MBC (μM)**						
		**005**	**B6**	**T6**	**L6**	**B11**	**T11**	**L11**
*E. coli*	ATCC 25922	2	2	0.5	4	1	2	2
*E. coli*	BW 25113	2	1	2	1	1	1	2
*E. coli*	BW 25113 Δ*sbmA*	2	2	2	2	1	2	2
*E. faecium*	ATCC 19434	>64	>64	>64	32	>64	32	16
*S. aureus*	ATCC 25923	16	16	32	8	32	16	8
*K. pneumoniae*	ATCC 700603	4	8	8	4	8	4	4
*A. baumannii*	ATCC 19606	4	2	2	2	2	2	2
*P. aeruginosa*	ATCC 27853	16	16	32	8	16	16	8
*E. cloacae*	ATCC 13047	16	4	4	2	4	4	2

^#^ The suffix B7- was omitted from all compound names for clarity. ^§^ MIC and MBC were recorded after 18 h of incubation at 37 °C. Results are the mode of at least three independent experiments (*n* = 3). The geometric mean of the same results is presented in Table S1. Coloured boxes indicate different antimicrobial activity: high activity (green) low or no activity (red).

## Data Availability

Data are freely available upon request to the corresponding authors.

## Supplementary Materials

The following supporting information can be downloaded at https://www.mdpi.com/article/10.3390/biom15060776/s1, Figure S1: Peptides precipitation assay. Table S1: Geometric mean of the MIC and MBC data.

## Abbreviations

The following abbreviations are used in this manuscript:

AMPs	Antimicrobial peptides
ANOVA	Analysis of variance
ATCC	American Type Culture Collection
CFU	Colony-forming unit
CLSI	Clinical and Laboratory Standards Institute
DMEM	Dulbecco’s modified Eagle’s medium
DMSO	Dimethyl sulfoxide
FBS	Fetal bovine serum
FluC	Firefly luciferase
GRAVY	Grand average of hydropathicity
IC_50_	Half maximal inhibitory concentration
MBC	Minimal bactericidal concentration
MHB	Mueller–Hinton broth
MIC	Minimum inhibitory concentration
MTT	3-(4,5-Dimethylthiazol-2-yl)-2,5-Diphenyltetrazolium bromide assay
OD	Optical density
PBS	Phosphate-buffered saline
PI	Propidium iodide
PrAMPs	Proline-rich antimicrobial peptides
RP-HPLC	Reverse Phase High Performance Liquid Chromatography
SD	Standard deviation
TFA	Trifluoroacetic acid
