# Supplementary material for: Designing New Chimeric Proline-Rich Antimicrobial Peptides to Enhance Efficacy Toward the ESKAPE+E: Beyond Sequence Extension
Source: Biomolecules. 2025 May 27;15(6):776. doi: 10.3390/biom15060776 (PMC12190468; doi:10.3390/biom15060776)
Supplement: Supplementary file 1 [file biomolecules-15-00776-s001.zip › biomolecules-3571313-supplementary.pdf]

Article

Designing New Chimeric Proline-Rich Antimicrobial Peptides to Enhance Efficacy Toward the ESKAPE+E: Beyond Sequence Extension

Adriana Di Stasi [1,†], Luigi de Pascale [1,†], Martino Morcici [2], Daniel N. Wilson [2], Marco Scocchi [1,*] and Mario Mardirossian [1,*]

[1] Department of Life Sciences, University of Trieste, 34127 Trieste, Italy; adriana.distasi@units.it (A.D.S.); luigi.depascale@phd.units.it (L.d.P.)
[2] Institute for Biochemistry and Molecular Biology, University of Hamburg, 20146 Hamburg, Germany; martino.morici@uni-hamburg.de (M.M.); daniel.wilson@uni-hamburg.de (D.N.W.)
* Correspondence: mscocchi@units.it (M.S.); mmardirossian@units.it (M.M.)
† These authors contributed equally to this work.

Supplementary materials

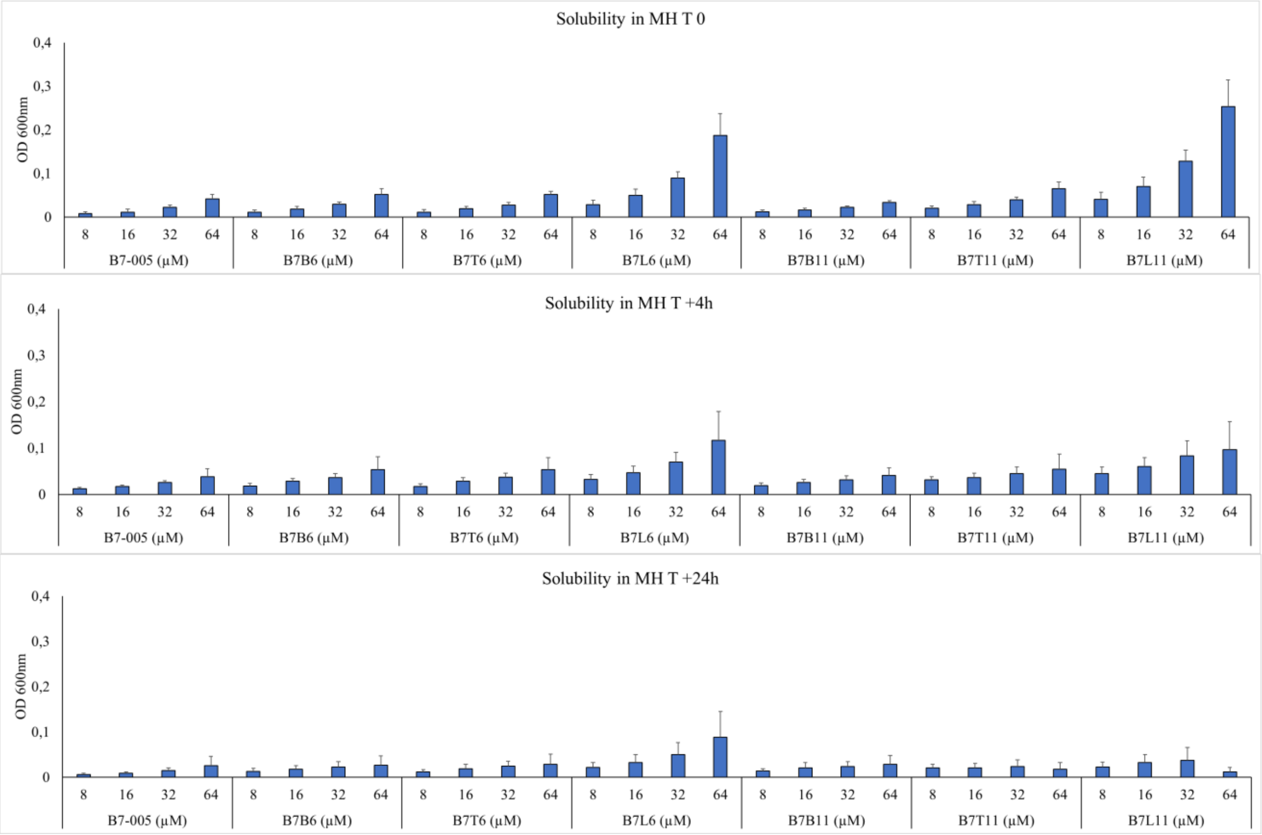

Figure S1. Peptides precipitation assay. Optical density (OD 600 nm) measurement of chimeric peptides solutions at different concentrations in MH broth incubated at 37°C up to 24 hours.

Table S1 Antimicrobial activity of B7-005 and its derivatives. Representative strains of the ESKAPE+E were used, as well as *E. coli* BW25113 and *E. coli* BW25113Δ*sbmA*.

Bacteria strain		MIC (µM)						
		**B7-005**	**B7B6**	**B7T6**	**B7L6**	**B7B11**	**B7T11**	**B7L11**
E.coli	ATCC 25922	1,6	1,6	0,6	3,2	0,8	1,3	2,5
E.coli	BW 25113	1	1	1	1	0,6	1	1,3
E.coli	BW 25113 ΔsbmA	1,6	1,6	1,3	1,6	1,0	1,3	1,6
E.faecium	ATCC 19434	25,4	10,1	16,0	4,0	16,0	5,0	4,0
S.aureus	ATCC 25923	16,0	10,1	16,0	8,0	20,2	12,7	5,0
K.pneumoniae	ATCC 700603	2,0	2,0	2,0	2,5	2,0	2,0	2,5
*A.baumanni*i	ATCC 19606	4,0	1,0	1,0	1,6	0,8	1,3	2,0
P.aeruginosa	ATCC 27853	20,2	10,1	12,7	5,0	12,7	12,7	6,3
E.cloacae	ATCC 13047	10,1	2,0	2,5	2,0	2,0	2,0	2,0

Bacteria strain		MBC (µM)						
		**B7-005**	**B7B6**	**B7T6**	**B7L6**	**B7B11**	**B7T11**	**B7L11**
E.coli	ATCC 25922	1,6	1,6	0,6	3,2	1,0	1,3	2,5
E.coli	BW 25113	2,5	2,0	2,5	1,6	1,0	1,3	1,6
E.coli	BW 25113 ΔsbmA	2,0	1,6	2,5	2,0	1,3	1,6	2,0
E.faecium	ATCC 19434	>64	>64	>64	40,3	>64	40,3	16,0
S.aureus	ATCC 25923	20,2	16,0	25,4	10,1	40,3	20,2	8,0
K.pneumoniae	ATCC 700603	5,0	8,0	8,0	3,2	6,3	3,2	3,2
A.baumannii	ATCC 19606	5,0	2,0	2,0	1,6	2,0	1,6	2,0
P.aeruginosa	ATCC 27853	25,4	16,0	25,4	8,0	20,2	20,2	8,0
E.cloacae	ATCC 13047	12,7	4,0	4,0	2,5	4,0	4,0	2,5

[#] The suffix B7- was omitted from all compound names for clarity.

[§] MIC and MBC were recorded after 18h of incubation at 37°C.

Results are the geometric mean of at least three independent experiments (n = 3).
